# Personals

**Published:** 1913-07

**Authors:** 


### PERSONALS.

*Announcements of removals, travel, and other matters of interest are requested. Please report any errors in the listing of any physician, in the State and other directories that we may co-operate with the State Society in securing a correct list of physicians.*

Dr. A. Crandall Way of Perry was the guest of the editor in June.

Dr. James A. Hawley, a graduate of the Eclectic Medical College of Pennsylvania in 1862, and an old resident of Canandaigua, has retired from active practice.

Dr. Montgomery E. Leary of Rochester, served as Surgeon-General in charge of the Hospital and Ambulance Corps, during the Elks' Convention in Rochester in June.

Dr. G. Jeanneny of France, whose valuable work on venous anaesthesia was abstracted in our February issue, has received the gold medal of the Bordeaux Medical Society in recognition of his researches.

Dr. G. G. Reynolds of Nichols, N. Y., will sail shortly for an extended European trip.

Dr. Roland Meisenbach of Buffalo has been elected a corresponding member of the St. Louis Medical Society.

Dr. Catherine E. Kelley of Buffalo has removed to 441 Fargo avenue.

Dr. George M. Lewis of Buffalo has removed to 2798 Delaware avenue, Kenmore.

Dr. Charles B. Tweedale of Cheboygan was the sole out-of-town representative of the class of 1888, Buffalo, to attend the 25th reunion. For this reason we have decided not to devote the space to a description of this reunion, for which we had an excellent apology prepared, believing that the personal interest which we felt in it demanded an apology.

Dr. Augustus F. G. Zurhorst has been appointed Postmaster at Oakfield, N. Y.

Dr. R. M. Root of Buffalo will sail for Europe this month.

Dr. R. R. Ross, Supt. of the Buffalo General Hospital, will spend July and August in Europe.

